# Study on Volatile Organic Compounds and Antioxidant Polyphenols in Cumin Produced in Xinjiang

**DOI:** 10.3390/ijms26062628

**Published:** 2025-03-14

**Authors:** Minghao Sun, Xufang Lv, Xiuxiu Liu, Wenyu Chen, Xing Shen, Zhongping Chai, Maomao Zeng

**Affiliations:** 1 College of Resources and Environment, Xinjiang Agricultural University, Urumqi 830052, China; sun18453303381@126.com (M.S.); 15035048162@163.com (X.L.); liuxiuxiu3629@163.com (X.L.); chenwenyu666666@163.com (W.C.); 2 Xinjiang Key Laboratory of Soil and Plant Ecological Processes, Xinjiang Agricultural University, Urumqi 830052, China; 3 State Key Laboratory of Food Science and Resources, Jiangnan University, Wuxi 214122, China; mmzeng@jiangnan.edu.cn; 4 School of Food Science and Technology, Jiangnan University, Wuxi 214122, China

**Keywords:** cumin, VOCs, polyphenolic compounds

## Abstract

This article investigated the composition and content of volatile organic compounds (VOCs) in cumin from three Xinjiang origins (Hami, Turpan, and Hetian) at different processing temperatures. VOCs varied with temperature and origin, but alcohols and terpenes were predominant in all samples. Hetian cumin exhibited the highest VOC content and stability under specific treatments, divided into an ambient temperature treatment (AMB) and a 70 °C heat treatment. A cluster analysis revealed high similarity between replicates and significant differences among the samples. A Venn diagram comparison showed that 70 °C processing reduced the number of common VOCs among the three origins from 36 to 19, which is a decrease of 47.22%, indicating a significant impact of heating on cumin VOCs and possibly promoting the formation of new compounds. Finally, utilizing the varying abilities of different types of polyphenols to inhibit heterocyclic aromatic amines (HAAs), six polyphenolic compounds, identified as sesamin, 6-caffeoylsucrose, apigenin, eschweilenol C, kaempferol glucuronide, and luteolin, were preliminarily determined to play an active role in the *β*-carboline HAA simulation system.

## 1. Introduction

Cumin is a classic spice with a strong, spicy, and distinctive aroma [1], commonly used in daily diets. Originating from Egypt and Ethiopia, cumin plays a significant role in the cuisines of various countries, such as Mexico, Thailand, Vietnam, and India, adding unique flavors to foods. Additionally, cumin has garnered attention for its medicinal properties, with its essential oils and extracts widely applied in the medical field for treating various ailments [2,3]. Usman [4] demonstrated that incorporating cumin into chicken nuggets extended their shelf life due to its antioxidant properties. Cumin is rich in nutrients and active substances, such as proteins, fats, polyphenols, and flavonoids. The active substances, such as polyphenols and flavonoids, possess antioxidant capabilities [5], scavenging free radicals, reducing oxidative stress, and enhancing immune function [6]. Furthermore, cumin essential oil has analgesic and anti-inflammatory properties [7,8]. In Xinjiang, due to its unique history and customs, cumin is extensively used, particularly in grilled mutton skewers and lamb chops, to enhance food aroma. With the increasing demand for cumin, its cultivation area and production in Xinjiang have been rising annually, providing a crucial economic source for local farmers and promoting regional economic development and rural revitalization. Therefore, studying cumin is of great significance.

Recently, as people’s living standards have improved, more attention has been paid to food health. Besides the nutritional value of food itself, cooking methods and storage conditions also significantly impact human health. Studies showed that high-temperature cooking could damage food DNA, potentially harming the consumer’s genome [9]. High-temperature frying affects protein and lipid oxidation in chicken breast meat and promotes the formation of advanced glycation end products (AGEs) [10], which are linked to inflammation, oxidative stress, and various chronic diseases. Additionally, thermal processing, like frying, can lead to the Maillard reaction, forming highly reactive carbonyl compounds, such as AGEs, heterocyclic aromatic amines (HAAs), acrylamide (AA), 5-hydroxymethylfurfural (5-HMF), and polycyclic aromatic hydrocarbons (PAHs). These compounds can react with others to form harmful substances, adversely affecting human health [11]. However, solutions may be found within foods themselves. Spices are known for their antimicrobial and antioxidant properties, and some can inhibit the formation of harmful compounds during food processing. For example, Zeng et al. found that rosemary essential oil effectively inhibits acrylamide formation during fried dried beef production [12]. Rosemary resin and sage oil resin exhibit strong antioxidant activity [13]. Other studies showed that turmeric, lemongrass, and galangal significantly reduced HAA formation in grilled beef patties, demonstrating good antioxidant properties [14]. Although research on spices is abundant, studies on cumin’s antioxidant function and deep components are relatively scarce. Thus, studying cumin not only meets the practical needs of Xinjiang but also has profound practical and significant value in promoting the scientific development of local specialty foods and enhancing public health.

Considering geographical location, soil conditions, and climatic features, this study selected cumin from Hami, Turpan, and Hetian in Xinjiang, representing the typical cumin-producing areas of southern and eastern Xinjiang. These regions are fertile and well-irradiated, producing high-quality cumin. By comparing VOC content, changes, and stability under AMB and a 70 °C heat treatment, we aimed to select the cumin variety with the most complex and rich aroma. Furthermore, we investigated polyphenolic compounds in VOCs through a qualitative analysis using a *β*-carboline HAA simulation system to identify the types of polyphenolic compounds that exhibit antioxidant properties and inhibit harmful substances. This study deepens our understanding of VOCs and polyphenolic compounds in cumin, providing a solid theoretical basis for scientific processing and improved cooking methods. By systematically analyzing these bioactive components, we lay a foundation for exploring cumin’s health benefits and potential applications in enhancing food quality, contributing to the healthy development of the cumin industry and advancements in food science.

## 2. Results and Discussion

### 2.1. Changes in VOC Content of Cumin from Different Origins Under Different Processing Temperatures

**Figure 1:**
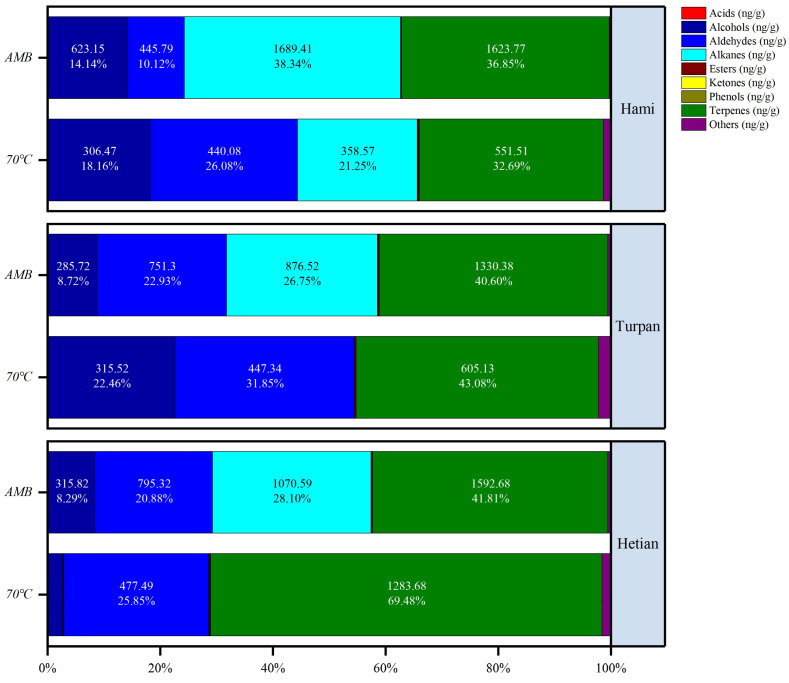
After treatment with AMB and 70 °C for 3 h, differences are seen in the percentage content of VOC categories in cumin produced in Hami, Turpan, and Hetian.

Figure 1 presents the distribution characteristics of volatile organic compound categories in cumin from the three production regions of Hami, Turpan, and Hotan after AMB treatment and treatment at 70 °C for 3 h. Upon data analysis, we found that geographical differences had a significant impact on the composition of cumin, a conclusion that aligns with the findings of Kesen et al [15]. Across all production regions, alcohols and terpenes were the main components of cumin.

Specifically, for cumin from Hami, under the 70 °C treatment, the terpene content was 551.51 ng/g, accounting for 32.69%, with an alkane content of 358.57 ng/g, accounting for 21.25%, and an alcohol content of 306.47 ng/g, accounting for 18.16%. Under the AMB treatment, the alkane content significantly increased to 1689.41 ng/g, accounting for 38.34%, while the terpene content was 1623.77 ng/g, accounting for 36.85%, followed by alcohols at 623.15 ng/g, accounting for 14.14%, and aldehydes at 445.79 ng/g, accounting for 10.12%. For cumin from Turpan, under the 70 °C treatment, the terpene content was the highest at 605.13 ng/g, accounting for 43.08%, with relatively high contents of alcohols and aldehydes at 22.46% and 31.85%, respectively, while the alkane content was extremely low at 0.35 ng/g, accounting for 0.02%. Under the AMB treatment, the terpene content remained the highest at 1330.38 ng/g, accounting for 40.60%, with the alkane content increasing to 876.52 ng/g, accounting for 26.75%, and aldehydes at 751.30 ng/g, accounting for 22.93%. For cumin from Hotan, under the 70 °C treatment, the terpene content was as high as 1283.68 ng/g, accounting for 69.48%, with aldehydes in second place at 477.49 ng/g, accounting for 25.85%, while the contents of alcohols and alkanes were both very low at 48.10 ng/g, accounting for 2.60%, and 0.70 ng/g, accounting for 0.04%, respectively. Under the AMB treatment, terpenes and alkanes jointly dominated, with contents of 1592.68 ng/g, accounting for 41.81%, and 1070.59 ng/g, accounting for 28.10%, respectively, followed by aldehydes at 795.32 ng/g, accounting for 20.88%, and alcohols at 315.82 ng/g, accounting for 8.29%.

In summary, regardless of the region or temperature treatment condition, terpenes and alcohols consistently remained the main volatile organic compound components in cumin [16]. The contents of esters, aldehydes, acids, and ketones were relatively low [15]. Particularly noteworthy is the unique change in alkane compounds, which exhibited extremely significant differences in content under different temperature treatments. Different treatment methods led to differences in the content of volatile organic compounds [17], with the AMB treatment resulting in the release of more volatile organic compounds, while the content of volatile organic compounds under the 70 °C treatment was relatively lower.

**Figure 2:**
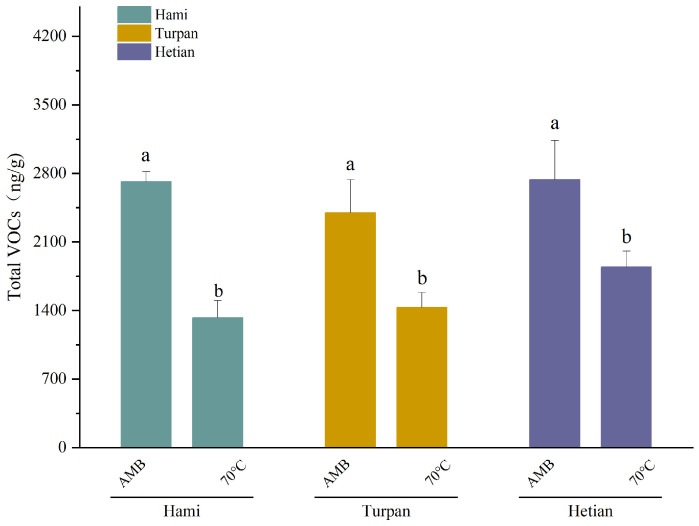
Total VOC values of cumin from different origins. Different lowercase letters in the same column indicate significant differences (*p* < 0.05).

From Table 1, it is evident that the processing temperature and production region had significant effects on the content of certain volatile organic compounds (VOCs) in cumin. On the one hand, the high-temperature treatment markedly reduced the content of key VOCs in cumin, which aligned with the overall trend observed in Figure 2. This is also highly consistent with the conclusions drawn by Kiralan et al. regarding the impact of heating methods on black cumin [17]. For instance, the content of 1,4-p-Menthadien-7-al. in Hami cumin decreased from 428.86 ± 186.31 µg/g during AMB to 243.83 ± 37.73 µg/g after treatment at 70 °C, representing a 44% reduction. Ma et al. also reached similar conclusions in their experiments involving the high-temperature heating of peanut oil [18]. 1,4-p-Menthadien-7-al, a monoterpene compound widely present in spice plants, such as cumin and mint, possesses a strong minty aroma and is one of the important flavor components in spices [19]. Additionally, it exhibits certain antimicrobial and antioxidant properties, enhancing the preservative capacity of spices. Similar trends are observed for *γ*-terpinene, cuminaldehyde, and limonene, which are also crucial flavor components in spices [20] and possess physiological properties, such as antioxidant activity [21].

Moreover, the relationships among VOCs were quite complex. Different types of VOCs not only had unique flavors and physiological properties but could also enhance the overall flavor and functional characteristics through synergistic and complementary effects. For example, cuminaldehyde, which underwent significant changes in content across different regions, contributed to the pungent and herbal aroma of cumin, while *γ*-terpinene and limonene, which were also present in high concentrations, contributed to the fresh citrusy and minty aroma. The combination of these compounds significantly enhanced the overall flavor profile of cumin, making it more complex and balanced. In terms of physiological properties, the synergistic effects among VOCs were equally notable. For instance, a study by Walid [22] found that there was a synergistic effect between the active components D-limonene and *γ*-terpinene in citrus essential oils, where their combined use exceeded the sum of their individual capabilities when used alone. We speculated that in the VOCs of cumin, *γ*-terpinene exerted its effects by scavenging free radicals, while cuminaldehyde enhanced antioxidant effects by inhibiting oxidase activity. The combined effect of the two may have been more pronounced than when used alone.

The changes in VOC content under different treatments suggested that high temperatures may have accelerated the volatilization or degradation of these heat-sensitive compounds. A similar phenomenon had been observed in the processing of dry-cured bacon [23], where high temperatures led to a decrease in VOC content, possibly due to oxidation or thermal decomposition. These changes directly affected the flavor and quality of cumin. VOCs are the main source of cumin’s aroma, and a reduction in their content weakens the aroma, thereby affecting its application in food. Therefore, optimizing the processing temperature is crucial for retaining the flavor characteristics of cumin and meeting the demand for high-quality spices in the food industry. On the other hand, despite being located in Xinjiang, the three production regions exhibited significant differences in VOC content in cumin due to variations in climate and soil conditions. This corroborates the findings of Kesen et al [15], which highlight that geographical origin is a significant factor influencing the volatile organic compounds (VOCs) in cumin. Furthermore, another study utilizing a GC-MS analysis of VOCs in different seed spices has also revealed that variations in origin significantly impact the proportions of aldehydes and terpenes, reinforcing the notion that geographical location plays a crucial role in determining the VOC composition of spices [16,19]. For example, the higher content of 1,4-p-Menthadien-7-al in Turpan may have been related to the region’s abundant sunshine, large diurnal temperature differences, soil fertility, and mineral content. Studies had shown that climatic conditions (such as temperature and light) could promote the synthesis and release of certain VOCs, while soil conditions (such as fertility and pH) could also affect their content. These changes not only influenced the flavor of cumin but also had significant implications on the physiological and ecological functions of the plant. Certain VOCs (such as terpenes) had defensive functions against pests and diseases, and changes in their content could affect the plant’s stress resistance. Furthermore, the release of VOCs was involved in signaling among plants, influencing the structure and function of plant communities. Therefore, by optimizing the planting environment (such as selecting appropriate production regions), the content of key flavor compounds in cumin could be increased, thereby improving its quality.

As can be seen in Figure 1 and Table 1, we found that the VOCs in cumin exhibited significant and unique variations under different environments, a phenomenon that was widespread and likely closely related to the quality of cumin. Cumin contained a variety of VOCs, including acids, alcohols, aldehydes, alkanes, esters, ketones, phenols, and terpenes. Under different production regions (Hami, Turpan, Hetan) and temperature conditions (AMB and 70 °C), the changes in the various types of compounds varied. For example, acids, with high chemical stability and conserved biosynthetic pathways, may have had limited direct impact on flavor formation. Acids such as acetic acid and 4-hydroxybutyric acid were only detected in small amounts at ambient temperatures in specific production regions. The chemical reactivity of alcohols was regulated by the environment; for instance, 1,4-Cyclohexadiene-1-methanol, 4-(1-methylethyl) decreased in content with the increasing temperature in the Hami region. Alkanes exhibited significant differences in content when the production region and temperature changed. Esters appeared or underwent significant changes in content under specific conditions. Notably, terpenes showed marked variations in content, high chemical reactivity, and finely tuned biosynthesis by the environment, making them highly likely to be key factors determining the flavor and quality of cumin. Going forward, we plan to delve into the specific mechanisms by which these VOCs contribute to the formation of cumin’s flavor and quality through sensory evaluation, quantitative-activity relationship studies of flavor compounds, and GC-O technology combined with sensory analyses, providing a scientific basis for the cultivation, processing, and utilization of cumin.

Due to the much smaller contribution of alkanes to aroma compared to other categories (alcohols, aldehydes, terpenes, acids, phenols), we excluded the alkanes and proceeded with the graphical comparison. As shown in Figure 2, there were differences in the total volatile organic compound (VOC) values of cumin from different origins. After the AMB treatment, the total VOC value of the Turpan group was the lowest at 2398.36 ng/g; in contrast, the total VOC values of cumin from Hami and Hetian were higher and essentially similar. For cumin treated at 70 °C, the total VOC values were generally lower than those treated with AMB. The total VOC value of the Hami group was the lowest at 1327.64 ng/g, while the total VOC value of the Hetian group was the highest at 1843.75 ng/g. It is noteworthy that the total VOC value of cumin from Hami decreased significantly after treatment at 70 °C, with the largest decrease of 51.11%. Meanwhile, the total VOC content of cumin from Hetian had the lowest decrease at only 32.61%, indicating the most stable chemical properties of cumin from Hetian. Based on a comprehensive consideration of various aspects, we will focus on investigating the polyphenolic antioxidants in cumin from Hetian in our subsequent studies.

### 2.2. Changes in VOC Types and Contents of Cumin from Different Origins Under Different Processing Temperatures

**Figure 3:**
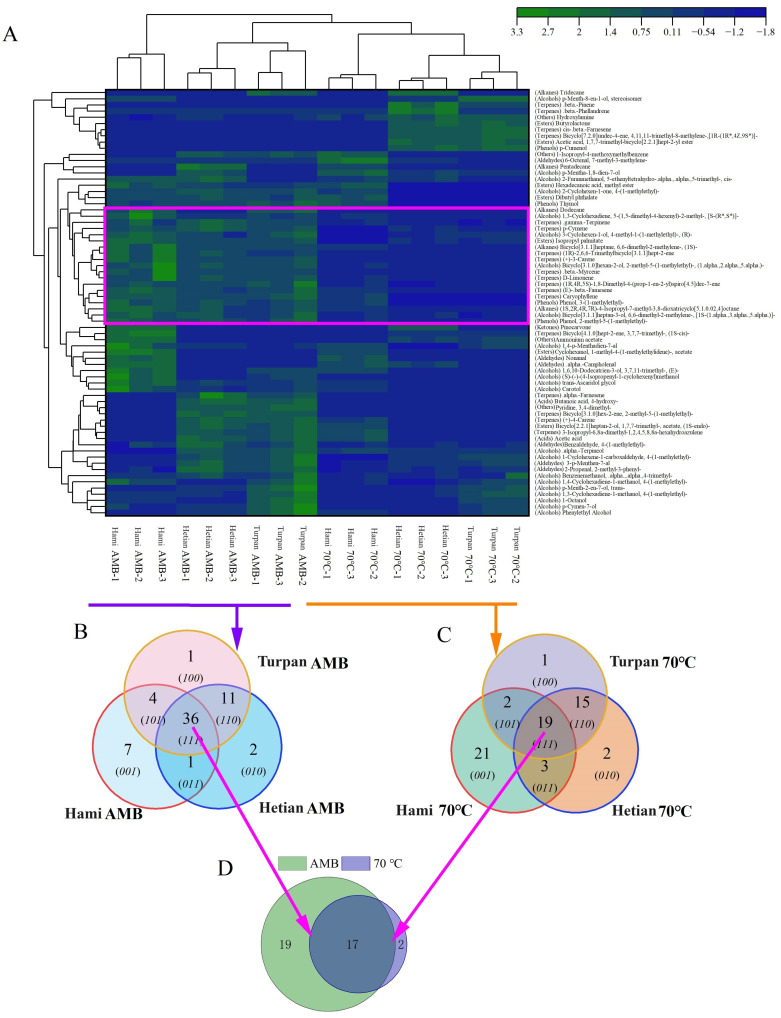
Analysis of VOCs in cumin from different origins under different processing conditions. (**A**) Cluster analysis. (**B**) Venn diagram of AMB heating conditions. (**C**) Venn diagram of heating conditions at 70 °C. (**D**) Venn diagram of common substances. The * indicates that the specific stereochemical configuration of the compound was not explicitly specified in its naming.

A cluster analysis (Figure 3A) showed that VOCs from the three origins clustered on the left under the AMB treatment but shifted to the right after the 70 °C heating. This suggests that some VOCs may undergo chemical changes or volatilization during heating, altering their clustering pattern at 70 °C. Heating may also promote chemical reactions such as hydroxyl radical [24] scavenging, cyclization, and decomposition [25,26], leading to the formation of new compounds. These new compounds may differ in chemical properties and volatility from original compounds, further contributing to the clustering shift at 70 °C. The red-boxed VOCs in Figure 3A show the most significant changes, with approximately half being terpenes, likely due to their lower molecular weights and higher volatility [27]. Terpenes are abundant in plants, especially aromatic ones, and are crucial for plant aroma and spices [28,29].

Comparing the common VOCs among the three origins under different processing conditions, we found that the 70 °C treatment reduced the number of shared VOCs by half, to only 19. This indicates that heating may cause the decomposition or volatilization of some VOCs. A Venn diagram comparison of 19 VOCs at 70 °C (Figure 3C) and 36 VOCs under the AMB treatment (Figure 3B) revealed that 17 compounds were stable and less affected by heating. Additionally, two new compounds were formed (Figure 3D), possibly due to heating-promoted chemical reactions. These results demonstrate that heating significantly affects the composition and content of VOCs in cumin, particularly volatile components, which is consistent with Ma’s findings on peanut VOCs [18]. Moreover, heating may promote the formation of new compounds, providing valuable insights into cumin quality and safety.

### 2.3. Differences in VOCs Under Different Treatment Conditions

In this study, lines were drawn to highlight the most color-distinct regions, clearly demonstrating the differences among the samples that were subjected to different temperature treatments. Notably, the region marked with a pink box exhibited the most significant differences under the two different temperature treatment conditions. Specifically, the content of 19 compounds in the samples treated at 70 °C showed a marked decrease compared to the AMB-treated group. It was worth noting that nearly two-thirds of these compounds belonged to the terpene class of VOCs. This phenomenon could be attributed to the high sensitivity of terpenes to temperature changes, as high temperatures might accelerate their degradation process and reduce their stability. This finding was highly consistent with the results of Xu’s study on the aroma changes in heat-treated blackberry clear juice using SPME-GC-MS technology [30].

Upon analyzing the 19 compounds enclosed in the box, it was found that many of them might contribute to food antioxidant properties. For instance, de Oliveira, TM found that p-Cymene exhibits significant antioxidant activity and might act as a neuro-protectant in the brain [31]. Similarly, *γ*-terpinene could reduce quinones to catechol, thereby exerting its antioxidant activity [32]. Additionally, 2-methyl-5-(1-methylethyl)-phenol had demonstrated significant antioxidant properties in mouse experiments [33]. Future research could focus on investigating the effects of these VOCs and their derivatives on the health benefits of cumin.

After exploring the changes in volatile organic compounds (VOCs) under different temperature treatment conditions, we further focused on polyphenols, which are also likely influenced by environmental factors, such as temperature. Polyphenols are a class of natural compounds commonly studied for their antioxidant and other health benefits in humans, and they are equally important secondary metabolites in plants, alongside VOCs. Studies have shown that there is an overlap in their composition, with certain VOCs serving as precursors to polyphenols; for example, acidic compounds, like gallic acid, are important constituents of polyphenol structures [34], and some monomeric lignins (such as p-coumaryl alcohol, coniferyl alcohol, and sinapyl alcohol) are the basic units of lignin biosynthesis [35]. Moreover, polyphenols and VOCs interact with each other, potentially inhibiting each other’s formation and functionality [36]. Therefore, studying the changes in polyphenols under different treatment conditions not only helped us to comprehensively understand the chemical composition of cumin but also enabled the dual optimization of cumin’s flavor and health benefits.

### 2.4. Identification of Polyphenolic Compounds in Hetian Cumin Extract

**Figure 4:**
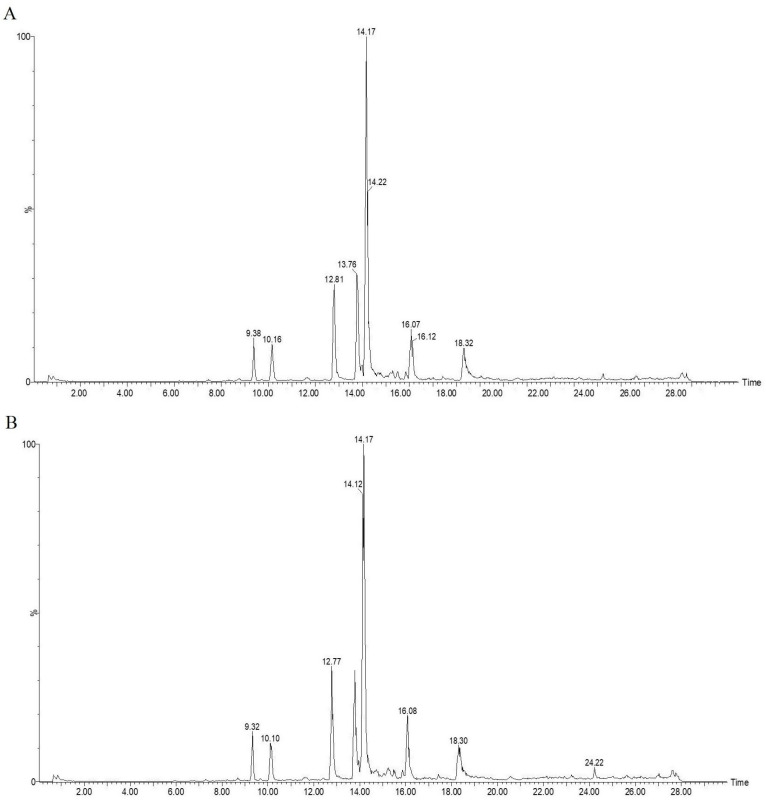

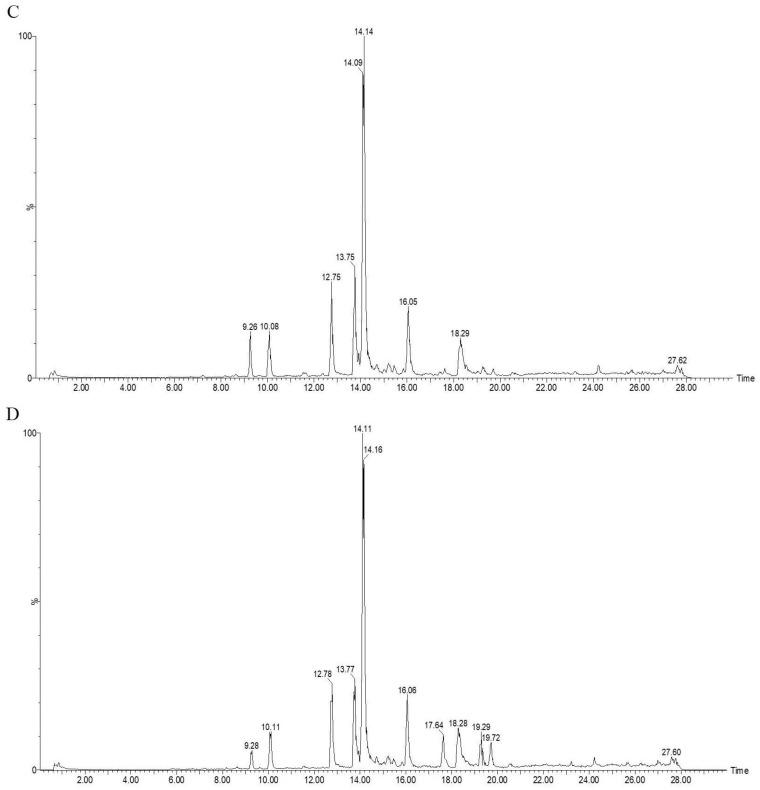
UV absorption spectra at 280 nm of cumin extract added to a *β*-carboline HAA model system at different heating temperatures. (**A**) AMB; (**B**) 70 °C; (**C**) 100 °C; (**D**) 120 °C.

Polyphenols are a class of compounds widely recognized as beneficial to human health. In this study, we conducted an in-depth exploration of their antioxidant properties and ability to inhibit harmful substances [37]. We used the precursors for HAA synthesis as substrates in the reaction system. Polyphenols can exert their effects by scavenging free radicals and blocking precursor reactions [14]. Therefore, we could preliminarily identify the types of polyphenols by observing their inhibitory effects on HAA formation and the resulting decrease in quantity. We used 0.1 g of freeze-dried powder of Hetian cumin ethanol extract, added it to the *β*-carboline HAA simulation system, and reacted it at AMB, 70 °C, 100 °C, and 120 °C for 3 h. UPLC-QTOF-MS was used to analyze the polyphenolic compounds in the extract, and peak areas of ten major peaks at different response times were integrated (Table 2) to study their changes. At 280 nm, ten prominent peaks appeared in the chromatograms (Figure 4) under different temperatures. Seven peaks showed decreasing peak areas with the increasing temperature, similar to Valois’s findings on the antioxidant activity of pyrolyzed bio-oil [38]. These results suggest that these polyphenolic compounds play an active role in the *β*-carboline HAA simulation system, exhibiting strong antioxidant properties.

**Figure 5:**
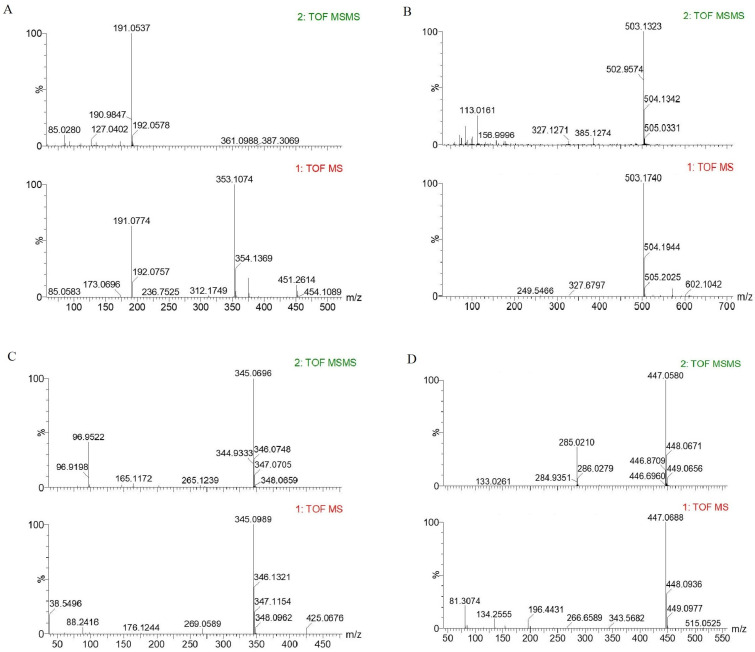

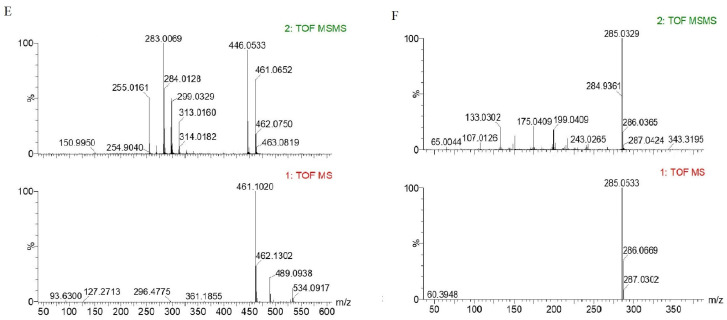
Mass spectrometry information for six polyphenolic substances extracted from Hetian cumin. (**A**–**F**) represent the components detected in Table 3, respectively. (**A**): Sesamin; (**B**): 6-Caffeoylsucrose; (**C**): Pobolin; (**D**): Eschweilenol C; (**E**): Kaempferol Glucuronide; (**F**): Luteolin.

Based on the information provided by the chromatograms, the primary and secondary mass spectrometry data of the seven polyphenolic compounds with decreasing peak areas were analyzed. Using the MassLynx mass spectrometry data analysis software (V4.2), the Progenesis QI LC/MS data analysis software (QI2.0), the ChemSpider database (2023), and MSFINDER data analysis software (V3.61), six potential polyphenolic compounds were identified in the extract of Hetian cumin. Specifically, based on the parent ion and daughter ion information (*m*/*z* = 353.1074 and *m*/*z* = 191.0537), as shown in Figure 5A, combined with the analysis data from various software and database comparisons, it was speculated that this peak might be sesamin. Similarly, based on the parent ion and daughter ion information in Figure 5B–F, along with the analysis data and database comparisons, it was speculated that these peaks might be 6-caffeoylsucrose, apigenin, eschweilenol C, kaempferol glucuronide, and luteolin, respectively (see Table 3). Among these, luteolin has been proven in previous studies to be an effective component in red peppers for inhibiting the formation of heterocyclic amines [39], and both sesamin and kaempferol glucuronide have been shown to have good antioxidant activities against lipid oxidation [40,41], capable of inhibiting harmful substances generated during high-temperature deep-frying processes. Spinach chlorophyll and its derivatives also exhibit antioxidant and anti-inflammatory effects [42]. However, the identification of these compounds was only determined based on their mass-to-charge ratios from mass spectrometry, so further confirmation is needed through isolation and purification, as well as comparison with chromatographic profiles of standard references. Additionally, there may be other polyphenolic flavonoids that have not yet been identified. Their functional potential aligns closely with the current research hotspot in flavonoid isomers. Isocoumarins, which share similar structures with the substances detected in the article and are flavonoid isomers (such as isocoumarin derivatives), exhibit significant antioxidant and anti-inflammatory activities due to minor structural differences (such as hydroxyl substitution sites or glycosylation patterns). This suggests that the incompletely characterized polyphenols in this study may encompass similar active isomers. The optimization of compound **1c** is a crucial direction. The dual inhibitory effect of compound **1c** on 5-LOX and PGE2 production indicates that it can be further optimized and tested in clinical trials for its potential in treating inflammatory diseases and cancer. Secondly, the new synthetic method for 3-glycosylated isocoumarins provides a foundation for exploring the biological activities of these compounds, with the prospect of developing new drugs with improved pharmacokinetic properties [43,44]. Furthermore, the synergistic effects of polyphenol compounds are also a worthy research direction. The polyphenol compounds identified in Hetian cumin (such as luteolin and sesamin) offer opportunities to study their synergistic effects with isocoumarin derivatives in anti-inflammatory and antioxidant stress responses.

## 3. Materials and Methods

### 3.1. Experimental Materials

Mature cumin seeds from Hami, Turpan, and Hetian in Xinjiang were collected and identified by Professor Zhongping Chai from the College of Resources and Environment, Xinjiang Agricultural University. After impurity removal, drying, and grinding, the cumin powder was stored at −20 °C until use. Chromatographic-grade formic acid and acetonitrile were used (TEDIA, Fairfield, CA, USA). Formic acid of chromatographic grade (Thermo Fisher, Waltham, MA, USA) and methanol and ethanol (analytical grade) (GuoYao, Shanghai, China) were used.

### 3.2. Instruments

The instruments used included an SCC61 E universal steam oven (Rational, Munich, Germany), SB-4200DTD ultrasonic cleaner (Scientz, Ningbo, China), Eppendorf 5425 high-speed centrifuge (Eppendorf, Hamburg, Germany), LGJ-25C freeze dryer (Sihuan, Beijing, China), Smart-S2-30DH ultrapure water system (Yipu Yida, Nanjing, China), AutoEva-60 automatic nitrogen concentrator, and Mem Spring nitrogen generator (Ruike, Xiamen, China). Additionally, an ultra-high-performance liquid chromatography quadrupole time-of-flight mass spectrometry system (Waters, Milford, MA, USA) and a BEH C18 column (100 mm × 2.1 mm i.d., 1.7 μm) (Waters, Milford, MA, USA) were employed.

### 3.3. Preparation of Cumin Ethanol Extract

The method by Yeoh and Ali [45] was slightly modified to prepare a cumin ethanol extract. Briefly, 2 g of cumin powder was mixed with 40 mL of 70% ethanol solution and subjected to ultrasonic-assisted extraction three times (30 min each at 30 °C). The combined extracts were centrifuged (4 °C, 4000 rpm, 10 min), and the supernatant was freeze-dried. The freeze-dried powder was redissolved in 4 mL of methanol and stored at −20 °C for later use.

### 3.4. Headspace Solid-Phase Microextraction

Based on the method of Rizzo et al. (2022) [46], GC-MS was used for a quantitative analysis of the volatile organic compounds in Xinjiang cumin. Firstly, 2 g of cumin powder was added to a 20 mL glass vial, followed by the addition of 20 μL of 2-methyl-3-heptanone methanol solution with a concentration of 118.2 μg/L as the internal standard. Prior to the extraction, the SPME fiber was conditioned at the GC inlet for 30 min at 270 °C. For the extraction, the sample vial was equilibrated at 50 °C for 30 min, after which the SPME fiber was inserted into the injection port of the GC-MS instrument and desorbed for 7 min at 250 °C with a splitless injection.

### 3.5. GC-MS Analysis Conditions

The GC conditions are as follows: An SH-WAX capillary column (30 m × 0.25 mm, 0.25 μm, Dao Jin, Kyoto, Japan) was used. The initial column temperature was set to 40 °C and held for 3 min, then ramped 6 °C/min to 100 °C, and subsequently 10 °C/min to 250 °C, where it was held for 5 min. The injection port temperature was 250 °C, and the detector temperature was also 250 °C. The carrier gas was high-purity He (purity > 99.99%) with a flow rate of 1 mL/min. The MS conditions are as follows: The ionization mode was EV with an electron energy of 70 eV, a filament emission current of 200 μA, an ion source temperature of 250 °C, and an interface temperature of 250 °C. The scan mass range was 35–450 *m*/*z*, and the detection voltage was 350 V.

### 3.6. Establishment of β-Carboline HAAs Simulation System

The *β*-carboline HAAs simulation system (Table 4) was used as the basis, with glucose, creatinine, and tryptophan added at 0.2 mmol, 0.4 mmol, and 0.4 mmol, respectively. Additionally, 0.1 g of freeze-dried powder of Hetian cumin ethanol extract was added to the system, which was then reacted at AMB, 70 °C, 100 °C, and 120 °C for 3 h. These concentrations were selected based on the relevant literature and experimental experience and optimized before the experiment.

### 3.7. Identification of Polyphenolic Compounds in the Simulation System 

The polyphenolic compound identification in the simulation system followed the methods by Xu et al. [47] and Pavlović et al. [48] with minor modifications. The total polyphenols extracted from the cumin samples were provided by Liu et al. [49]. After centrifugation (4 °C, 10,000 rpm, 10 min), 1 mL of the reaction solution was analyzed using UPLC-QTOF-MS. The elution conditions were as follows: 0–5 min, 5–10% A; 5–10 min, 10–25% A; 10–15 min, 25–50% A; 15–20 min, 50–80% A; 20–25 min, 80–100% A; 25–30 min, 5–10% A. A BEH C18 column (100 mm × 2.1 mm i.d., 1.7 μm) was used with a column temperature of 45 °C, an injection volume of 10 μL, and a detection wavelength range of 200–600 nm. The mobile phase consisted of pure acetonitrile (A) and a 0.1% formic acid aqueous solution (B) at a flow rate of 0.3 mL/min. The mass spectrometry conditions were set to an ESI ionization source, dual-channel negative ion mode, mass-to-charge ratio of 50–1500, collision energy of 6.0 eV (channel 1) and 20 eV (channel 2), capillary voltage of 3.0 kV, cone voltage of 30.0 V, cone gas flow rate of 10 L/h, desolvation gas flow rate of 700 L/h, ion source temperature of 100 °C, and desolvation gas temperature of 400 °C.

### 3.8. Statistical Analysis

The UPLC-MS/MS quantitative results were analyzed using the MassLynx V4.1 software (Waters, Milford, MA, USA). Data were subjected to a one-way ANOVA with a completely randomized design using Statistix 9.0 (Analytical Software, Tallahassee, FL, USA), followed by a Tukey HSD test for multiple comparisons (*p* = 0.05). Data are presented as mean ± standard deviation (*n* = 3). HAA data were imported into SIMCA 14.1 (Umetrics, Umea, Sweden) for a PCA analysis. Spearman’s rank correlation was assessed using Origin Pro 2021b (OriginLab, Northampton, MA, USA). The qualitative results were compared with the mass spectra in the WILEY9 database. Additionally, the retention indices were calculated using a mixture of n-alkanes ranging from C8 to C26 as standards, and these indices were then compared with the Kovats’ indices (KIs) in the NIST17 database to perform a qualitative analysis of the compounds. Graphs were plotted using the Origin Pro 2021b and the Anaconda Jupyter Notebook V6.4.8 (Anaconda, Austin, TX, USA).

## 4. Conclusions

This study investigated the composition and content of volatile organic compounds (VOCs) in cumin from different geographical origins under varying temperatures. The results indicated that there are differences in the VOC composition of cumin from different regions, with alcohols and terpenes being the primary components. Furthermore, the temperature treatment during processing was found to affect the content and composition of VOCs, with the AMB treatment resulting in the release of more VOCs. Additionally, the study revealed that the processing may facilitate chemical reactions, leading to the formation of new compounds.

Using the freeze-dried powder of an ethanol extract from Hetian cumin as the research subject, it was added to a simulated system of *β*-carboline heterocyclic amines (HAs). By reacting at different temperatures, polyphenolic compounds with inhibitory effects on *β*-carboline HAAs were identified. The results showed that, after integrating the peak areas of ten major peaks observed at different response times, the peak areas of seven peaks decreased with the increasing reaction temperature. These polyphenolic compounds were analyzed using primary and secondary mass spectrometry for compound identification. Ultimately, six potential polyphenolic compounds were identified in the Hetian cumin extract, including sesamin, 6-caffeoylsucrose, apigenin, eschweilenol C, kaempferol glucuronide, and luteolin. It should be noted that these identifications are only preliminary hypotheses and require further experimental validation and confirmation. Additionally, there may be other unidentified compounds that necessitate a more in-depth analysis and identification.

## Figures and Tables

**Table 1:** The content of VOCs in cumin from different origins varies with different processing temperatures.

Number	Name		Hami AMB µg/g	Hami 70 °C µg/g	Hetian AMB µg/g	Hetian 70 °C µg/g	Turpan AMB µg/g	Turpan 70 °C µg/g
1	Butanoic acid, 4-hydroxy-	Acids	n.d.	n.d.	1.17 ± 0	n.d.	1.11 ± 0.24	n.d.
2	Acetic acid	Acids	n.d.	0.69 ± 0.14	0.55 ± 0.48	n.d.	0.88 ± 0.22	n.d.
		**Total**	n.d.	0.69 ± 0.14	1.72 ± 0.48	n.d.	1.99 ± 0.46	n.d.
3	1,4-p-Menthadien-7-al	Alcohols	428.86 ± 186.31	243.83 ± 37.73	178.46 ± 21.96	n.d.	109.83 ± 20.79a	239.95 ± 25.14b
4	Bicyclo[3.1.0]hexan-2-ol, 2-methyl-5-(1-methylethyl)-, (1.*α*.,2.*α*.,5.*α*.)-	Alcohols	143.9 ± 57.76a	41.53 ± 6.07b	97.9 ± 13.46a	23.26 ± 2.31b	90.85 ± 16.94a	14.67 ± 2.11b
5	p-Cymen-7-ol	Alcohols	12.1 ± 5.46	8.8 ± 1.2	14.04 ± 2.53a	7.83 ± 1.1b	34.4 ± 10.27	18.74 ± 2.02
6	p-Menth-2-en-7-ol, *trans*-	Alcohols	3.45 ± 0.33	3.28 ± 0.47	8.61 ± 1.55a	5.37 ± 0.76b	21.35 ± 7.21	13.62 ± 1.67
7	Bicyclo[3.1.1]heptan-3-ol, 6,6-dimethyl-2-methylene-, [1*S*-(1.*α*.,3.*α*.,5.*α*.)]-	Alcohols	4.74 ± 0.47a	2.62 ± 0.32b	4.33 ± 0.75a	2.61 ± 0.32b	4.26 ± 1.21	2.51 ± 0.32
8	3-Cyclohexen-1-ol, 4-methyl-1-(1-methylethyl)-, (*R*)-	Alcohols	8.98 ± 0.89	n.d.	5.46 ± 2.32	2.58 ± 0.94	8.04 ± 2.47a	3.93 ± 0.66b
9	1,4-Cyclohexadiene-1-methanol, 4-(1-methylethyl)-	Alcohols	9.5 ± 3.17	4.44 ± 0.46	3.5 ± 0.68a	1.91 ± 0.33b	11.36 ± 3.64a	7.13 ± 0.83b
10	.*α*.-Farnesene	Alcohols	n.d.	n.d.	0.54 ± 0.26	n.d.	0.31 ± 0	n.d.
11	2-Furanmethanol, 5-ethenyltetrahydro-.*α*.,.*α*.,5-trimethyl-, *cis*-	Alcohols	0.45 ± 0a	0.24 ± 0b	0.51 ± 0.1a	0.25 ± 0b	n.d.	n.d.
12	Benzenemethanol, *.α*.,.*α*.,4-trimethyl-	Alcohols	0.45 ± 0	0.37 ± 0	0.54 ± 0.1a	0.34 ± 0b	0.59 ± 0.2	0.49 ± 0.24
13	p-Mentha-1,8-dien-7-ol	Alcohols	n.d.	0.52 ± 0.1	0.42 ± 0a	0.22 ± 0b	n.d.	0.21 ± 0
14	1,3-Cyclohexadiene-1-methanol, 4-(1-methylethyl)-	Alcohols	0.44 ± 0	n.d.	0.42 ± 0.1	0.33 ± 0	1.66 ± 0.44	1.29 ± 0.2
15	*trans*-Ascaridol glycol	Alcohols	0.96 ± 0.24a	0.25 ± 0b	0.35 ± 0	n.d.	0.34 ± 0.1	n.d.
16	1,3-Cyclohexadiene, 5-(1,5-dimethyl-4-hexenyl)-2-methyl-, [*S*-(R*,S*)]-	Alcohols	1.35 ± 0.71a	0.17 ± 0.1b	0.31 ± 0.1	n.d.	0.51 ± 0.1	n.d.
17	Carotol	Alcohols	0.85 ± 0.36a	0.32 ± 0b	0.31 ± 0.1a	0.13 ± 0b	0.39 ± 0.1a	0.19 ± 0b
18	Phenylethyl Alcohol	Alcohols	n.d.	0.08 ± 0	0.12 ± 0	n.d.	0.45 ± 0.14a	0.17 ± 0b
19	p-Menth-8-en-1-ol, stereoisomer	Alcohols	5.71 ± 0.32	n.d.	n.d.	3.32 ± 0.37	n.d.	12.22 ± 0.39
20	1-Octanol	Alcohols	0.21 ± 0	n.d.	n.d.	n.d.	0.92 ± 0.22a	0.4 ± 0b
21	1,6,10-Dodecatrien-3-ol, 3,7,11-trimethyl-, (*E*)-	Alcohols	0.05 ± 0a	0.03 ± 0b	n.d.	n.d.	0.03 ± 0	n.d.
22	(*S*)-(−)-(4-Isopropenyl-1-cyclohexenyl)methanol	Alcohols	1.15 ± 0.36	n.d.	n.d.	n.d.	0.43 ± 0.14	n.d.
		**Total**	623.15 ± 141.79a	306.47 ± 46.44b	315.82 ± 43.21a	48.16 ± 5.86b	285.72 ± 63.67	315.52 ± 32.12
23	Nonanal	Aldehydes	0.53 ± 0.1	0.38 ± 0.1	n.d.	n.d.	n.d.	n.d.
24	.*α*.-Campholenal	Aldehydes	0.22 ± 0	0.14 ± 0	n.d.	0.1 ± 0	n.d.	n.d.
25	3-p-Menthen-7-al	Aldehydes	80.27 ± 2.92	78.8 ± 15.24	168.04 ± 23.41a	112.08 ± 11.01b	184.19 ± 50.11	124 ± 14.67
26	6-Octenal, 7-methyl-3-methylene-	Aldehydes	0.34 ± 0a	3.26 ± 0.46b	0.44 ± 0a	0.2 ± 0b	0.24 ± 0	n.d.
27	Benzaldehyde, 4-(1-methylethyl)-	Aldehydes	355.96 ± 118.03	357.18 ± 54.28	596.57 ± 83.92a	347.05 ± 33.99b	527.31 ± 139	326.2 ± 37.69
28	1-Cyclohexene-1-carboxaldehyde, 4-(1-methylethyl)-	Aldehydes	8.08 ± 0.92	n.d.	29.33 ± 4.73a	17.58 ± 2.8b	38.67 ± 11.69	26.61 ± 3.58
29	2-Propenal, 2-methyl-3-phenyl-	Aldehydes	0.4 ± 0.14	0.32 ± 0.1	0.94 ± 0.14a	0.49 ± 0b	0.9 ± 0.22a	0.53 ± 0b
		**Total**	445.79 ± 119.93	440.08 ± 69.99	795.32 ± 112.26a	477.49 ± 47.66b	751.3 ± 200.87	477.34 ± 55.82
31	Bicyclo[3.1.1]heptane, 6,6-dimethyl-2-methylene-, (1*S*)-	Alkanes	1687.35 ± 635.85a	357.67 ± 46.72b	1069.14 ± 50.97		874.69 ± 131.91	n.d.
32	(1*S*,2*R*,4*R*,7*R*)-4-Isopropyl-7-methyl-3,8-dioxatricyclo[5.1.0.02,4]octane	Alkanes	1.4 ± 0.17a	0.61 ± 0.1b	1.01 ± 0.22a	0.37 ± 0b	0.86 ± 0.3	0.35 ± 0
33	Pentadecane	Alkanes	n.d.	0.29 ± 0	0.44 ± 0.35	n.d.	n.d.	n.d.
34	Dodecane	Alkanes	0.67 ± 0.3	n.d.	n.d.	n.d.	0.65 ± 0	n.d.
35	Tridecane	Alkanes	n.d.	n.d.	n.d.	0.33 ± 0b	0.32 ± 0	n.d.
		**Total**	1689.41 ± 635.49a	358.57 ± 46.84b	1070.59 ± 50.98a	0.7 ± 0b	876.52 ± 132.22a	0.35 ± 0b
36	Bicyclo[2.2.1]heptan-2-ol, 1,7,7-trimethyl-, acetate, (1*S*-endo)-	Esters	n.d.	3 ± 0.14	5.38 ± 0.87	n.d.	5.6 ± 1.62	n.d.
37	Isopropyl palmitate	Esters	1.9 ± 0.41	n.d.	1.23 ± 0.2a	0.58 ± 0b	1.25 ± 0.28a	0.6 ± 0b
38	Hexadecanoic acid, methyl ester	Esters	0.21 ± 0	0.19 ± 0	0.13 ± 0	n.d.	0.12 ± 0	n.d.
39	Butyrolactone	Esters	n.d.	n.d.	n.d.	0.39 ± 0.1	n.d.	0.41 ± 0.1
40	Acetic acid, 1,7,7-trimethyl-bicyclo[2.2.1]hept-2-yl ester	Esters	n.d.	n.d.	n.d.	2.15 ± 0	n.d.	2.18 ± 0.1
41	Cyclohexanol, 1-methyl-4-(1-methylethylidene)-, acetate	Esters	0.77 ± 0.14a	0.23 ± 0b	n.d.	n.d.	n.d.	
		**Total**	2.88 ± 0.58	3.42 ± 0.17	6.74 ± 1.09a	3.11 ± 0.17b	6.97 ± 1.91a	3.19 ± 0.2b
42	Pinocarvone	Ketones	1.61 ± 0			1.4 ± 0	n.d.	n.d.
43	2-Cyclohexen-1-one, 4-(1-methylethyl)-	Ketones	1.75 ± 0.14	1.59 ± 0.2	1.67 ± 1.12		2.16 ± 0.39	n.d.
		**Total**	3.36 ± 0.14a	1.59 ± 0.2b	1.67 ± 1.12	1.4 ± 0	2.16 ± 0.39	n.d.
44	Hydroxylamine	Others	13.93 ± 3.69a	22.73 ± 4.37b	19.27 ± 2.48a	29.09 ± 4.66b	17.14 ± 3.2a	30.24 ± 1.3b
45	1-Isopropyl-4-methoxymethylbenzene	Others	n.d.	0.26 ± 0	0.18 ± 0	n.d.	0.18 ± 0	n.d.
46	Ammonium acetate	Others	1.05 ± 0	n.d.	n.d.	0.43 ± 0.1	n.d.	0.38 ± 0.1
47	Pyridine, 3,4-dimethyl-	Others	n.d.		0.89 ± 0	n.d.	0.92 ± 0.1	
		**Total**	14.98 ± 3.68	22.99 ± 4.4	20.33 ± 2.52a	29.52 ± 4.77b	18.23 ± 3.23a	30.62 ± 1.38b
48	Phenol, 3-(1-methylethyl)-	Phenols	0.99 ± 0.22a	0.47 ± 0b	0.78 ± 0	n.d.	0.66 ± 0.2	
49	Phenol, 2-methyl-5-(1-methylethyl)-	Phenols	0.84 ± 0.14a	0.28 ± 0b	0.67 ± 0.14a	0.12 ± 0b	0.76 ± 0.36a	0.22 ± 0b
50	Thymol	Phenols	0.11 ± 0	0.14 ± 0	0.11 ± 0	n.d.	0.18 ± 0	n.d.
51	p-Cumenol	Phenols	n.d.	n.d.	n.d.	0.26 ± 0	n.d.	0.29 ± 0
		**Total**	1.93 ± 0.35a	0.88 ± 0.1b	1.56 ± 0.17a	0.39 ± 0b	1.6 ± 0.6a	0.51 ± 0b
	.*γ*.-Terpinene	Terpenes	710.86 ± 176.97	430.93 ± 64.05	849.95 ± 99.06a	416.68 ± 4.12b	664.54 ± 47.39a	271.54 ± 97.75b
52	p-Cymene	Terpenes	284.75 ± 6.24	n.d.	336.14 ± 37.96a	134.43 ± 14.1b	299.92 ± 57.11a	99.82 ± 10.23b
53	.*β*.-Myrcene	Terpenes	304.76 ± 146.99a	47.94 ± 6b	183.25 ± 21.33a	51.08 ± 4.85b	176.09 ± 27.77a	33.39 ± 3.34b
54	(1*R*)-2,6,6-Trimethylbicyclo[3.1.1]hept-2-ene	Terpenes	219.63 ± 78.01a	26.09 ± 3.68b	133.97 ± 18.17	n.d.	89.87 ± 10.28	n.d.
55	d-Limonene	Terpenes	49.5 ± 26.68a	13.13 ± 2.5b	35.39 ± 3.87a	11.06 ± 0.1b	32.76 ± 8.18a	7.64 ± 1.68b
56	(1*R*,4*R*,5*S*)-1,8-Dimethyl-4-(prop-1-en-2-yl)spiro[4.5]dec-7-ene	Terpenes	13.89 ± 1.57a	6.96 ± 1.14b	11.98 ± 1.39a	4.1 ± 0.63b	16.45 ± 3.72a	5.77 ± 0.61b
57	(+)-4-Carene	Terpenes	n.d.	3.95 ± 0.62	8.08 ± 1.15	n.d.	8.09 ± 1.59	n.d.
58	(*E*)-.*β*.-Famesene	Terpenes	14.88 ± 0.98a	5.36 ± 0.94b	9.95 ± 2.71	n.d.	12.66 ± 3.38	n.d.
59	(+)-3-Carene	Terpenes	10.16 ± 3.92a	1.38 ± 0.14b	3.34 ± 2.51	n.d.	6.96 ± 0.99	n.d.
60	Caryophyllene	Terpenes	5.11 ± 0.59a	2.71 ± 0.33b	3.71 ± 0.51	n.d.	4.74 ± 1.19	n.d.
61	Bicyclo[3.1.0]hex-2-ene, 2-methyl-5-(1-methylethyl)-	Terpenes	n.d.	n.d.	0.17 ± 0	n.d.	0.15 ± 0	n.d.
62	Bicyclo[4.1.0]hept-2-ene, 3,7,7-trimethyl-, (1*S*-*cis*)-	Terpenes	10.25 ± 0.28	n.d.	n.d.	2.43 ± 0.2	n.d.	2 ± 0.24
63	Bicyclo[7.2.0]undec-4-ene, 4,11,11-trimethyl-8-methylene-,[1*R*-(1R*,4*Z*,9S*)]-	Terpenes	n.d.	n.d.	n.d.	2.35 ± 0.2	n.d.	2.98 ± 0.57
64	*cis*-.*β*.-Farnesene	Terpenes	n.d.	n.d.	n.d.	3.47 ± 0.66	n.d.	4.81 ± 0.61
65	.*β*.-Phellandrene	Terpenes	n.d.	n.d.	n.d.	325.02 ± 22.06	n.d.	21.36 ± 2.9
66	.*β*.-Pinene	Terpenes	n.d.	n.d.	n.d.	333.06 ± 67.13	n.d.	155.84 ± 23.19
67	3-Isopropyl-6,8a-dimethyl-1,2,4,5,8,8a-hexahydroazulene	Terpenes	n.d.	13.06 ± 2.17	16.21 ± 2.57	n.d.	17.83 ± 5.15	n.d.
68	.*α*.-Farnesene	Terpenes	n.d.	n.d.	0.54 ± 0.26	n.d.	0.31 ± 0	n.d.
		**Total**	1623.77 ± 129.16a	551.51 ± 79.41b	1592.68 ± 184.39	1283.68 ± 102.39	1330.38 ± 132.92a	605.13 ± 116.14b
		**ALL Total**	4405.3 ± 600.07a	1686.21 ± 246.01b	3806.44 ± 383.4a	1844.45 ± 151.42b	3274.88 ± 532.56a	1432.67 ± 162.43b

The “n.d.” stands for not detected. The * indicates that the specific stereochemical configuration of the compound was not explicitly specified in its naming. The data are presented as mean ± standard deviation (*n* = 3), with a significance level of *p* = 0.05. Different lowercase letters within the same row indicate significant differences (*p* < 0.05).

**Table 2:** Changes in the peak area of polyphenolic substances in cumin extract with temperature variation.

Peak Number	Response Time	AMB	70 °C	100 °C	120 °C
1	9.38	264	250	227	102
2	10.16	338	323	326	262
3	12.81	856	845	584	603
4	13.76	950	907	768	585
5	14.17	3557	3487	3252	2869
6	16.07	632	605	583	498
7	17.62	55	70	75	252
8	18.32	674	650	609	477
9	19.26	0	0	141	208
10	19.66	0	0	55	182

**Table 3:** Six polyphenolic compounds in Hetian cumin extract were found to have inhibitory effects on *β*-carboline HAAs.

Peak Number	Parent Ion (*m*/*z*)	Daughter Ion (*m*/*z*)	Molecular Formula	Compound
1	353.1074	191.0537	C_20_H_18_O_6_	Sesamin
2	503.1740	113.0161	C_21_H_28_O_14_	6-Caffeoylsucrose
3	345.0989	96.9522	C_17_H_14_O_8_	Pobolin
4	447.0688	285.2010	C_20_H_16_O_12_	Eschweilenol C
5	461.1020	283.0069	C_21_H_18_O_12_	Kaempferol Glucuronide
6	285.0533	133.0302	C_15_H_10_O_6_	Luteolin

**Table 4:** *β*-Carboline HAA-mixed aldehyde analogue system.

Reaction Precursor	Addition Amount
Glucose (mmol/mL)	0.02
Creatinine (mmol/mL)	0.04
Tryptophan (mmol/mL)	0.04
Cumin Ethanol Extract (g/mL)	0.01

## Data Availability

The original contributions presented in this study are included in the article. Further inquiries can be directed to the corresponding authors.
